# Androgen Deprivation Therapy, Hypogonadism and Cardiovascular Toxicity in Men with Advanced Prostate Cancer

**DOI:** 10.3390/curroncol28050289

**Published:** 2021-08-28

**Authors:** Gabriela Silvia Gheorghe, Andreea Simona Hodorogea, Ana Ciobanu, Ioan Tiberiu Nanea, Andrei Cristian Dan Gheorghe

**Affiliations:** 1 Faculty of Medicine, Department 4, Cardio-Thoracic Pathology, Carol Davila University of Medicine and Pharmacy, 050471 Bucharest, Romania; gabriela.gheorghe@umfcd.ro (G.S.G.); ana.ciobanu@umfcd.ro (A.C.); tiberiu.nanea@umfcd.ro (I.T.N.); acd2403@yahoo.com (A.C.D.G.); 2 Department of Internal Medicine and Cardiology, Theodor Burghele Clinical Hospital, 050653 Bucharest, Romania

**Keywords:** androgen deprivation therapy, hypogonadism, cardiovascular, thrombotic risk, arrhythmia, QT interval, prostate cancer

## Abstract

Androgen deprivation therapy (ADT) is successfully used in patients with advanced prostatic cancer, but there are many concerns about its systemic side effects, especially due to advanced age and frequent comorbidities in most patients. In patients treated with ADT there are metabolic changes involving the glycaemic control and lipid metabolism, increased thrombotic risk, an increased risk of myocardial infarction, severe arrhythmia and sudden cardiac death. Still, these adverse effects can be also due to the subsequent hypogonadism. Men with heart failure or coronary artery disease have a lower level of serum testosterone than normal men of the same age, and hypogonadism is related to higher cardiovascular mortality. Many clinical studies compared the cardiovascular effects of hypogonadism post orchiectomy or radiotherapy with those of ADT but their results are controversial. However, current data suggest that more intensive treatment of cardiovascular risk factors and closer cardiological follow-up of older patients under ADT might be beneficial. Our paper is a narrative review of the literature data in this field.

## 1. Introduction

Prostate cancer is the second most common cancer in men, accounting for 15% of all causes of cancer, 31.1 cases per 100,000 men and a mortality rate of 7.8 per 100,000 cases in 2012 [1]. It occurs especially in patients over 66 years [1] and is androgen dependent.

**Figure 1:**
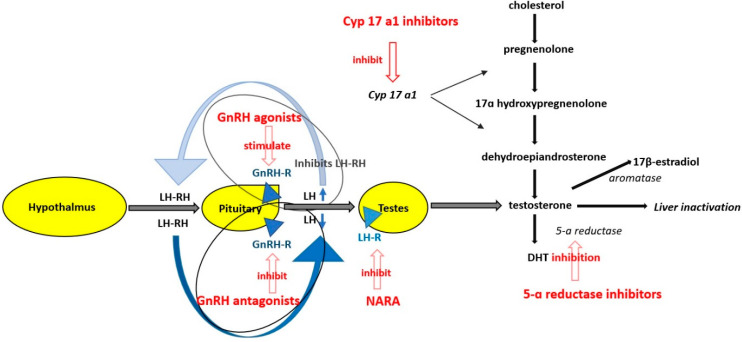
Mechanism of action of antiandrogen therapy GnRH = gonadotropin releasing hormone receptor agonist; LH = luteinizing hormone; LH-RH= luteinizing hormone releasing hormone; GnRH-R = gonadotropin releasing hormone receptor; GnRH antagonist = gonadotropin releasing hormone antagonist; LH-R = luteinizing hormone receptor on testis; NARA = nonsteroidal androgen receptor antagonis; DHT = dihidrotestosterone, CYP 17A1 = cytochrome P450 17 A1; 5–10% of testosterone is transformed by 5-α reductase in the more potent DHT, 0.2% testosterone is transformed by aromatases in 17β-estradiol and the rest of testosterone is inactivated by the liver; GnRH agonists provoke an increase of LH in the first weeks of treatment but finally down regulate LH secretion from the anterior pituitary gland. GnRH antagonists block the GnRH receptors on the anterior pituitary gland thus decreasing the synthesis of LH and FSH. NARA block the activation of the androgen receptor from the prostate cells and augment the effectiveness of GnRH agonists or antagonists, CYP 17 A1 inhibitors decrease the synthesis of testosterone from cholesterol.

Testosterone and its more potent derivative dihydrotestosterone (DHT) are involved in the maturation of the prostate. The normal serum level of testosterone in an adult male is more than 300 ng/dL [2] and 5–10% of testosterone is transformed by 5-α reductase in the more potent DHT, 0.2% of testosterone is transformed by aromatases in 17β-estradiol and the rest is inactivated by the liver (Figure 1) [3].

Experimental data suggest that androgens and the polymorphisms in the androgen signaling pathway promote tumorigenesis in animal models, and tumor regression is seen upon androgen deprivation [4,5]. The low level of serum testosterone can be achieved by bilateral orchiectomy, radiotherapy or androgen deprivation therapy (ADT) which is indicated in locally advanced disease (defined by T3-T4), N1, metastatic disease, non-metastatic castration-resistant disease and high-risk localized disease (defined by T3a or Gleason Score 8–10 or PSA (Prostate-specific antigen) > 20 ng/mL) [6].

There are several classes of ADT, according to their mechanisms of action (Figure 1). Synthetic estrogens are no longer used because of their adverse effects. The other classes of drugs are gonadotropin-releasing hormone receptor agonists (GnRH agonists), gonadotropin-releasing hormone receptor antagonists (GnRH antagonists), cytochrome P450 17 A1 (CYP 17A1) inhibitors, nonsteroidal androgen receptor antagonists and 5α-reductase inhibitors (Table 1). GnRH agonists provoke an increase of luteinizing hormone level (LH) and testosterone serum level in the first weeks of treatment but finally downregulate LH secretion from the anterior pituitary gland and decrease testosterone serum level. GnRH antagonists block the GnRH receptors on the anterior pituitary gland thus decreasing the synthesis of LH and of follicle-stimulating hormone (FSH) and finally the serum level of testosterone [5]. Nonsteroidal androgen receptor antagonists block the activation of the androgen receptor from the prostate cells and augment the effectiveness of GnRH agonists or antagonists [5]. CYP 17 A1 inhibitors decrease the synthesis of testosterone from cholesterol. In the strict sense, ADT includes only GnRH agonists and GnRH antagonists, but in many studies, this term also includes CYP 17 A1 inhibitors and nonsteroidal androgen receptor antagonists which are administrated in association with GnRH agonists and antagonists. Serum testosterone level usually decreases to <50 ng/dL under ADT.

The widespread use of these drugs improved the prognosis of prostate cancer [6] and many studies demonstrated a longer metastasis-free or overall survival period in patients who received ADT. On the other hand, there is a concern about the cardiotoxicity of ADT. Many data suggest that patients with prostate cancer who receive ADT have a higher cardiovascular risk than patients in whom hypogonadism is induced by orchiectomy or radiotherapy alone. These deleterious effects are more frequently encountered in the elderly in which the aging process itself leads to cardiovascular alterations related to the accumulation of epigenetic changes.

**Figure 2:**
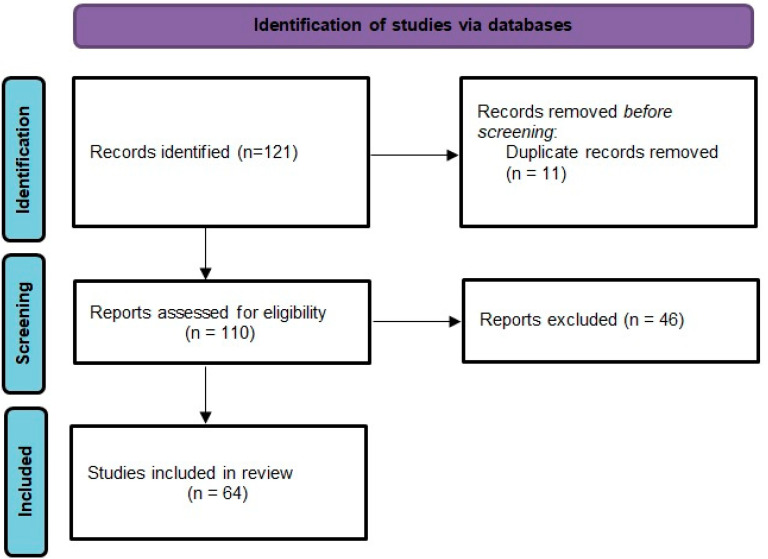
PRISMA 2020 flow diagram for the current review.

We conducted a narrative review of the literature on cardiovascular effect of testosterone, hypogonadism and ADT. We searched original articles, systematic reviews and meta-analyses published between 2006–2021 regarding the cardiovascular toxicity of the androgen deprivation therapy indexed in databases PubMed and Google Scholar and also by hand searching additional articles that were cited in the reference lists. The selected articles were all written in English, and we retained only those that were clear, precise. We assessed full articles, extracted relevant data and we removed duplicates. We selected observational studies, randomized controlled trials, meta-analyses that evaluated the cardiovascular effects of ADT by clearly defining the exposure to ADT, the comparison group, fatal and nonfatal cardiovascular outcomes (Table 2). The selection process is presented in a Preferred Reporting Items for Systematic Reviews and Meta-analyses (PRISMA) flow diagram [7] (Figure 2).

## 2. Cardiovascular Effects of Testosterone

There is a dual relationship between testosterone serum level and cardiovascular system function.

### 2.1. Favourable Effects

Among favorable cardiovascular actions of testosterone are antiarrhythmic effects, experimental coronary vasodilatation and reduced carotid artery intima-media thickness. Experimentally, endogenous testosterone limits coronary neointima formation after percutaneous coronary dilatation in male Yucatan miniature swine [8]. Testosterone shortens QTc interval on Electrocardiogram (ECG) and ventricular action potential duration. In addition, testosterone protects from severe ventricular tachycardia by decreasing the myocardial cells L-type calcium channel current and increasing several K currents occurring during phase 3 of the action potential: rapidly activating delayed rectifier current (I_kr_), slowly activating delayed rectifier current (I_ks_) and inward rectifier current (I_k1_) [9,10]. After orchiectomy, the QT interval becomes longer than in healthy age-matched male subjects.

Testosterone increases catecholamine-induced lipolysis, reduces lipoprotein lipase activity and triglyceride uptake in human abdominal adipose tissue, and decreases visceral fat accumulation thereby increasing lean mass and improving fasting glucose level [11,12]. Testosterone impairs human adipose stem cell commitment to preadipocytes through bone morphogenetic protein 4 (BMP4). Inhibitory effects of testosterone are mediated in part by androgen receptor action. Chazenbalka et al. [13], Rosen et al. [14], Madsen et al. [15] studied the effects of testosterone on subcutaneous adipose stem cells and demonstrated that the impairment of preadipocytes formation is performed by BMP4-induced nuclear hormone receptor peroxisome proliferator-activated receptor γ (PPARγ) and CCAAT/enhancer-binding protein C/EBPα mRNA expression An additional inhibitory effect of testosterone on early-stage preadipocyte differentiation and C/EBPβ mRNA expression may occur through the Janus kinase signal transducer and activator of transcription (STAT)3 signaling and JAK2/STAT3 pathway. Testosterone augmented cholesterol efflux from human monocyte–derived macrophages via upregulation of scavenger receptor B1 and can reduce the cholesterol content of atherosclerotic lesions [16,17].

### 2.2. Deleterious Effects

There are data confirming that testosterone has also deleterious cardiovascular effects. Testosterone induces cardiac hypertrophy and fibrosis, potentiates angiotensin II-induced renal vasoconstriction, reduces endothelium-dependent brachial artery dilatory capacity, causes low serum level of high-density lipoprotein (HDL cholesterol), increases the expression of pro-atherogenic genes, increases atheroma plaque volume, coronary calcifications, lipid loading of the macrophages and adherence of white blood cells to the endothelial cells [18].

### 2.3. Controversial Effects

Testosterone has controversial effects on the other lipoprotein particles, inflammatory markers, haemostasis [11,12]. Some data demonstrate lower levels of total serum cholesterol, LDL cholesterol and triglycerides in patients with normal testosterone levels [18]. Testosterone increases the level of the anti-inflammatory interleukine-10 (IL-10), decreases the level of proinflammatory interleukine-6 (IL-6), tumor necrosis factor α (TNF-α), interleukin -1 β (IL-1 β), interferon-γ (IFN-γ) but there are controversial effects on C- reactive protein (CRP) and vascular cell adhesion molecule-1 (VCAM) serum levels. Testosterone stimulates tissue factor pathway inhibitor (TFPI) and tissue plasminogen activator (tPA) expression, inhibits plasminogen activator inhibitor-1 (PAI-1) secretion, but, on the other hand, increases thromboxane A2 (TxA2) level, human platelets receptor density and platelets aggregation [19,20]. Some studies document vasodilation of the brachial artery after testosterone infusion [21]

## 3. Clinical Cardiovascular Effects of Hypogonadism

Despite the controversial experimentally data regarding the cardiovascular effects of testosterone, clinical data demonstrated that natural occurring or therapeutically induced hypogonadism in men has deleterious cardiovascular effects by worsening the risk factor profile or by direct action on the cardiovascular system.

### 3.1. Hypogonadism and Cardiovascular Risk Factors

There is a direct link between type 2 diabetes mellitus and hypogonadism [21,22]. Hypogonadism raises the risk of developing metabolic syndrome and type 2 diabetes mellitus. On the other hand, patients with type 2 diabetes mellitus have a low level of serum testosterone and testosterone replacement therapy was associated with a significant reduction in fasting plasma glucose, HbA1c, fat mass and triglycerides, as the meta-analysis performed by Corona et al. showed [12]. Obesity is associated with hypogonadism, as shown by the Swedish MrOS study [23] which performed an analysis on 2416 men, and testosterone replacement therapy improves body mass index. There could be several links between obesity and low serum testosterone level. Obesity can induce hypogonadism by increasing the aromatase level in adipose tissue and the conversion of testosterone into estradiol. The reduced tissue sensibility to insulin in type 2 diabetes mellitus can impair the capacity of the hypothalamus to secrete gonadotropin-releasing hormone and can induce hypogonadotropic hypogonadism [12] Moreover, obesity-associated hyperinsulinemia can reduce sex hormone-binding globulin [24] and consequently, the total testosterone serum level. On the other hand, experimental studies in mice demonstrate that low testosterone serum levels may augment the effects of a hypercaloric diet and promote obesity [24]. Tsai et al. [25] demonstrated in 110 s-generation Japanese American men that lower baseline testosterone level independently predicted an increase in intraabdominal fat after 7.5 years of follow-up. There are controversial data about the influence of hypogonadism on cholesterol lipoprotein serum level [21]. Endothelium-dependent brachial artery dilatory capacity, which is a marker of endothelial function, is increased in hypogonadal men but without known clinical implications [18].

### 3.2. Hypogonadism and QTc Interval on ECG

After orchiectomy, the QTc interval on ECG becomes longer than in healthy age-matched male subjects [26] Giraldi et al. [27] studied 26 men (mean age 39.2 ± 2.17 years) with pituitary or testicular hypogonadism compared with 26 age-matched control men and found a prolongation of QTc in 4 patients with hypogonadism and none in the normal group. However, they concluded that the differences are not relevant. On the other hand, Charbit et al. [28] studied QT interval changes in 11 hypogonadal men who received intramuscular testosterone and found a statistically significant reduction of 13.6 ± 2.8 ms between low and high levels of serum testosterone (363 ms versus 352 ms, mean QT value at a low and a high level of testosterone, respectively, *p* = 0.0001). Salem et al. [29] evaluated 7 men with torsade de pointes and demonstrated that all of them had low serum testosterone levels.

### 3.3. Hypogonadism and Cardiovascular Mortality

There are data that men with heart failure or coronary artery disease had a lower level of serum testosterone than normal men at the same age and that hypogonadism is related to higher cardiovascular mortality [21]. Haring et al. [30] found in 1954 men aged 20 to 79 years that a low serum testosterone level was significantly associated with total and cardiovascular mortality. The same results were reported by Menke et al. [31] in 1114 patients, during 9 years of follow-up, Vikan et al. [32] in 1568 patients, Shores et al. [33] in 858 patients, Tivesten et al. [34] in 3014 patients. However, Khaw et al. [35] found no statistically significant differences in cardiovascular and total mortality according to the serum testosterone level in 11606 men with cardiac pathology aged 40 to 79 years, despite noting a trend of higher mortality in hypogonadal patients. Some studies also reported survival improvement with testosterone replacement therapy [34].

## 4. Cardiovascular Effects of ADT

### 4.1. Mechanisms of Cardiovascular Effects of ADT

**Figure 3:**
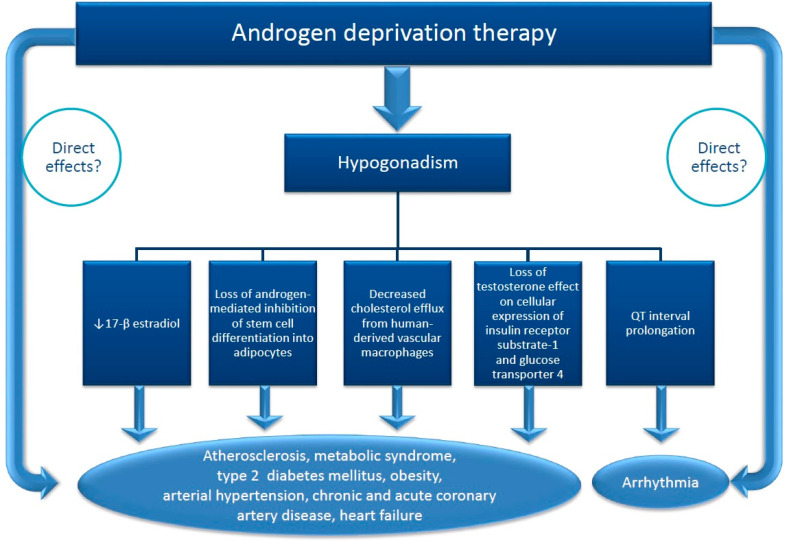
Supposed mechanisms of cardiovascular toxicity of androgen deprivation therapy (ADT) in patients with prostate cancer. Hypogonadism induced by ADT is involved in the occurrence of the cardiotoxicity by many mechanisms. The direct contribution of ADT is not obvious excepting CYP 17 inhibitors which indirectly induce a surge of mineralocorticosteroid secretion and GnRH agonists.

The above data demonstrated that hypogonadism, regardless of its cause, is associated with increased risk factors for cardiovascular diseases, severe ventricular arrhythmia and increased cardiovascular mortality. It is not clear whether the cardio-vascular deleterious effects of ADT are due only to induced hypogonadism or also to other mechanisms (Figure 3). ADT increases the risk of atherosclerotic events by inducing hypogonadism that leads to a decreased level of 17β-estradiol and decreased cholesterol efflux from human-derived vascular macrophages [16,17]. There is also an increase in insulin resistance, fasting glucose, leptin, HbA1c due to loss of the effect of testosterone on cellular expression of insulin receptor substrate-1 and glucose transporter 4 [16,36,37]. The adipose tissue accumulates mainly subcutaneously without changes in waist-hip ratio. ADT can also induce fatal ventricular arrhythmia by lengthening Q-T interval on ECG [38,39,40] due to hypogonadism but also by a direct effect on I_k_ currents in myocardial cells, described experimentally for some ADT [38] (Figure 3). ADT raises the thrombotic risk by increasing the serum level of fibrinogen, as demonstrated by Ziaran et al. [41] in 97 patients with locally advanced prostate cancer after 12 months of treatment, but not the serum level of CRP [16,42]. ADT increases the stiffness of the arterial wall but only abiraterone, which indirectly stimulated the secretion of mineralocorticoids hormones, and enzalutamide have been shown to induce arterial hypertension [15].

ADT is related to the prolongation of QT interval on ECG and to the occurrence of torsade de pointes or other severe ventricular arrhythmias [29,37]. According to Salem et al. [10], the GnRH antagonist degarelix and the CYP17 inhibitor abiraterone, which induces hypermineralocorticoidism and hypokalaemia, are more prone to induce a 10–20 ms prolongation of QT interval on ECG and torsade de pointes, compared to other ADT agents. However, experimental studies showed that degarelix did not affect hERG gene and membrane K channels, which are myocardial cell targets of drugs that prolong QT interval. The prolongation of the QT interval may be due to the hypogonadism induced by the drug [43]. At the same time, an experimental study in zebrafish showed that enzalutamide associated with terfenadine decreased heart rate and increased mortality in a dose-dependent manner [44].

### 4.2. Clinical Studies

Many studies compared the cardiovascular effects of ADT with those of hypogonadism induced by orchiectomy or radiotherapy, but their conclusions are not concordant. ADT was significantly associated with more non-fatal CV disease and stroke in comparison with no therapy (watchful waiting attitude), but not all studies demonstrated significant differences between ADT and orchiectomy and radiotherapy regarding cardiotoxicity [16]. These discrepancies can be explained by the different design of studies, duration of ADT administration, demographic data of patients included in the studies. The age of patients is important because aging induced biological changes [45,46,47,48] and concomitant diseases such as diabetes mellitus, chronic kidney disease, obesity, that can contribute to cardiovascular impairment. D’Amico et al. [49] analysed the prostate cancer, cardiovascular and overall mortality over a 16.62-year follow-up period in 206 men with unfavourable-risk prostate cancer randomized to receive radiotherapy alone or radiotherapy and 6 months of ADT. There was a significant increase in overall and cardiac mortality in patients with moderate or severe comorbidities randomized to radiotherapy and ADT in contrast to patients with no or minimal cardiac comorbidities. These differences appeared after 7.6 years from randomization. Morgans et al. [50] demonstrated that the risk of diabetes mellitus or cardiovascular disease in men under ADT for more than 2 years is increased in older but not in younger men and that these diseases occur after 5–10 years. Yamazaki et al. [51] studied other causes of mortality than prostate cancer in 1125 patients with localized disease treated with high-dose radiotherapy with and without ADT during a follow-up period of 80.7 months. They demonstrated that adding ADT to radiotherapy for more than 2 years increases the risk of other causes of death, especially in patients aged ≥75 years. The most frequent causes of death were the occurrence of other neoplasia and cerebrovascular diseases. However, there are studies that demonstrate the occurrence of cardiovascular toxicity of ADT in the first 6 months of treatment in patients with underlying cardiovascular comorbidities [52].

Table 3 summarizes the most important studies regarding the cardiotoxicity of ADT according to their results.

#### 4.2.1. Studies Showing an Additional Cardiovascular Risk in Patients on ADT

SEER/Medicare (Surveillance Epidemiology and End Results) study included 73,196 men over 66 years old who were diagnosed with locoregional prostate cancer between 1992 to 1999 and observed through 2001, treated with orchiectomy associated or not with GnRH agonist. Patients receiving GnRH agonists had significantly more non-fatal and fatal cardiovascular events (coronary heart disease, myocardial infarction, sudden cardiac death or life-threatening ventricular arrhythmias) compared to patients without GnRH agonists. These events occurred earlier in patients treated with than in those without GnRH agonists. Patients treated with orchiectomy alone were more likely to develop diabetes but not coronary heart disease, myocardial infarction or sudden cardiac death [53,66].

CaPSURE registry (Cancer of the Prostate Strategic Urologic Research Endeavor) analyzed 7248 men with prostate cancer divided in 4 groups: patients receiving ADT alone, patients with ADT and local therapy, patients with local therapy only and a group of patients with watchful/waiting/active surveillance. The primary endpoints were cardiovascular mortality, prostate cancer related mortality and all-cause mortality Patients treated with ADT alone had a 2-fold increase in cardiovascular mortality compared to patients treated only with local therapy. The authors considered the results inconclusive because of other unmeasured variables affecting treatment selection. The cardiovascular risk of patients under ADT was higher in those with a previous history of myocardial infarction, coronary artery disease or heart failure [59].

1372 men were enrolled in three randomized trials between February 1995 and June 2001 and randomly assigned to receive radiation therapy with or without ADT for 3, 6 or 8 months. There was a 2 year earlier occurrence of fatal myocardial infarction in patients over 65 years old treated for 6 months with ADT compared to patients of the same age without ADT and to patients less than 65 years old, irrespective of treatment [54].

O’Farrell et al. [52] studied 41,362 patients with prostate cancer-treated with ADT or orchiectomy in comparison with 187,785 men without prostate cancer. They found that the risk of cardiovascular disease was increased by 21% in patients who received ADT and by 16% in patients treated with orchiectomy compared to men without prostate cancer. The risk was higher in patients with a previous history of cardiovascular disease and increased in the first 6 months of treatment.

A 3.8-year follow-up of 1015 patients with prostate cancer treated with local therapy with or without ADT found an increased risk of sudden cardiac death in patients with ADT after 1–4 months of treatment [55].

Alibhai et al. [56] observed for a mean of 6.47 years 19,079 men over 66 with advanced prostate cancer treated with orchiectomy and ADT, matched with 19,079 men with the same diagnosis without ADT. They reported that patients who received ADT had a higher risk of diabetes and fragility fracture but not of acute myocardial infarction or sudden cardiac death.

According to CredibleMeds, among androgen receptor inhibitors, apalutamide prolongs the QT interval and can induce torsade de pointes.

In a recent study [39] which included 35 patients with advanced prostate cancer and secondary hypogonadism induced by 6 months ADT, there was a significant alteration of ECG parameters associated with arrhythmic risk: prolongations of QT interval corrected to the cardiac rate, QT dispersion, maximal value of Tpeak-Tend interval, mean ratio Tpe/QT and maximal ratio Tpe/QT, Tped. In patients which undergone an echocardiographic study there was also a subclinical alteration of global longitudinal strain and mechanical dispersion evaluated by echocardiography [67]. However, there is not clear if these effects are due to the ADT itself or to the induced hypogonadism. The concomitant use of ADT with CYP17 inhibitor abiraterone increases arrhythmic risk due to the association of hypokalaemia induced by abiraterone and prolongation of QT interval [42].

#### 4.2.2. Studies Showing No Additional Cardiovascular Risk in Patients on ADT

Nanda et al. [57] examined all-cause death in 5077 patients, median age of 69.5 years, with localized or locally advanced prostate cancer treated or not for 4 months with adjuvant ADT followed by radiotherapy. They found no difference in all-cause mortality between the two groups (9.6% in patients on ADT without comorbidities versus 6.7%, in patients without adjuvant ADT, *p* = 0.86). There were also no differences regarding all-cause mortality in patients with a single coronary artery disease risk factor on ADT and patients not receiving ADT (10.7% versus 7.0%, *p* = 0.82). However, there was a higher mortality rate in the subgroup of patients with pre-existing coronary artery disease treated with ADT compared to patients without adjuvant ADT (26.3% versus 11.2%, respectively, *p* = 0.04). The median follow-up time was 5.1 years in patients with adjuvant ADT and 4.4 years in the group without adjuvant ADT.

Radiation Therapy Oncology Group (RTOG) 92-02 [60] trial included 1554 patients with locally advanced prostate cancer treated for 4 months with radiotherapy and GnRH agonist followed or not by 24 months of adjuvant GnRH agonist. The cardiovascular mortality was greater in older patients with a history of cardiovascular disease and diabetes mellitus but was not influenced by the GnRH agonist treatment.

1113 patients with locally advanced prostate cancer received external-beam radiotherapy plus 6 months or 2.5 years of GnRH agonist. Adverse events included fatigue, diminished sexual function and hot flushes. There were not significant differences in fatal cardiac events between the two groups [58].

#### 4.2.3. Studies Comparing the Cardiovascular Risk of Various ADT

Moreira et al. [63] performed a meta-analysis concerning the cardiotoxicity of abiraterone-prednisone versus placebo-prednisone and enzalutamide versus placebo. There were 2283 patients in the arm abiraterone-placebo and 2914 patients in the arm enzalutamide-placebo. Abiraterone was associated with an increased risk of cardiovascular events, while enzalutamide was associated with an increased risk of fatigue.

Liang et al. [61] published a meta-analysis regarding ADT and the risk of cardiovascular disease and found an association between ADT and the occurrence of myocardial infarction but not of sudden cardiac death. The risk is increased in patients receiving abiraterone and enzalutamide. However, they did not find an association between the duration of ADT and the occurrence of myocardial infarction.

Hu et al. [16] found in 3 meta-analyses of observational studies a positive association between ADT and the occurrence of cardiovascular events, cardiovascular death and myocardial infarction. These associations were not always significant. On the other hand, 3 randomized control trials did not describe an association between ADT and cardiovascular outcomes except nonfatal cardiovascular disease. GnRH agonists have the most important cardiovascular adverse effects and the association with androgen receptor antagonists increased the risk of fatal and nonfatal cardiovascular events. GnRH antagonists have a better cardiovascular safety profile than GnRH agonists.

A meta-analysis that included 8660 patients with prostate cancer demonstrated that abiraterone significantly increase the risk of both cardiac toxicity and hypertension comparing to control, whereas enzalutamide significantly increases the risk of hypertension [68].

In the phase III HERO trial, which included 934 patients treated with GnRH agonist leuprolide or GnRH antagonist relugolix, this last one was associated with a 54% lower risk of nonfatal myocardial infarction, nonfatal stroke and all-cause mortality [62].

A phase II randomized, open-label study monitored 80 patients with advanced prostate cancer and pre-existing cardiovascular disease for one year. 41 patients received GnRH antagonist degarelix and 39 patients GnRH agonist. The primary endpoint was the endothelial function, and the secondary outcome was the occurrence of cardiovascular events. There were no differences between the GnRH agonist and GnRH antagonist arms regarding the endothelial function but there were fewer cardiovascular and cerebrovascular events on the GnRH antagonist compared to the GnRH agonist. 20% of patients randomized to GnRH agonist had a major cardiovascular and cerebrovascular event compared to 3% of those on GnRH antagonist (*p* = 0.013). The absolute risk reduction in major cardiovascular and cerebrovascular events at 12 months using GnRH antagonist was 18.1% (95% CI 4.6–31.2, *p* = 0.032) [64].

Zhang et al. [65] published recently a comparative cardiovascular risk profile of available ADT, by analyzing the FDA Adverse Events Reporting System (FAERS). They reviewed retrospectively the cardiovascular adverse effects which occurred in patients with prostate cancer treated with GnRH agonists, GnRH antagonists, androgen receptor antagonists and/or androgen synthesis inhibitors between January 2000 and April 2020. They reported 12.6% cardiovascular adverse events in patients on ADT monotherapy, especially GnRH agonists, and 26.1% in patients on combination therapy. The most frequent adverse effects were arterial vascular events, such as coronary artery disease and myocardial infarction, then arrhythmias, heart failure, venous thromboembolism. Second-generation androgen receptor antagonists and abiraterone monotherapy were associated with fewer myocardial infarctions and coronary artery disease.

## 5. Therapeutic Consequences

ADT is very useful in the treatment of advanced prostate cancer and usually, the side effects do not preclude its use. The cornerstone of the management of cardiovascular side effects of ADT is prevention. First it is very important to identify the patients at risk for the occurrence of cardiovascular side effects. These are the elderly with previous cardiovascular disease. Nguyen et al. [69] published a retrospective analysis of outcomes in 14,594 men, median age 71.8, with prostate cancer, treated with brachytherapy ± supplemental external beam radiation and followed up for 4.3 years. 1378 (9.4%) of patients had a history of chronic heart failure or myocardial infarction, 42.9% of them receiving a median of 4 months of ADT. There was an increase of all-cause mortality in patients with previous heart disease who received ADT. It is important to perform a complete cardiologic evaluation before initiating ADT especially in patients with previous heart disease. Cardiological treatment must be optimized to obtain the recommended targets of serum lipids, glucose metabolism, arterial blood pressure values, cardiac electrical and echocardiographic parameters.

The optimization of the cardiological treatment. The intense surveillance of the cardiac status of the patients is particularly important for the prevention of the occurrence of cardiovascular deleterious events. Some authors proposed an “ABCDE” paradigm concerning the control of risk factors for cardiovascular diseases [16,70]: Awareness and taking Aspirin, controlling Blood pressure and Cholesterol, stopping Cigarettes, controlling Diabetes and Exercise. Statin use for cardiovascular purposes can also improve the outcome of prostate cancer by reducing the captation of dehydroepiandrosterone in prostatic cancer cells, as shown in an experimentally study [71]. There are studies that demonstrated the cardiovascular benefits of regular physical activity in patients with prostate cancer on ADT. Culos-Reed et al. [72] demonstrated in 31 men, average age of 67 years, with localized or metastatic prostate cancer undergoing ADT that a 12-week home-based physical activity improve the quality of life. A meta-analysis published by Abdalla Ali Deb et al. [61] found that regular physical activity can prevent prostate cancer in normal men and improve the outcome of ADT in patients who already have the disease. Patients with prostate cancer under ADT, especially the elderly with a history of cardiovascular diseases, must be frequently followed up and their cardiovascular complications treated according to the existing guidelines.

## 6. Conclusions

ADT and subsequent hypogonadism are lifesaving in patients with advanced prostate cancer, but many studies describe cardiovascular adverse effects of this therapy. The elderly are more susceptible to the cardio-vascular toxicity of ADT. The comorbidities increase the cardiovascular risk of the prostate cancer patients under ADT. Various classes of ADT have different cardio-vascular adverse effects, and the risk is higher in combination therapy. There are no concluding data about the relationship between the duration of therapy and the occurrence of cardiotoxicity. The cardiotoxicity can occur in the first 6 months of treatment but also later, after years of follow-up. A systematic cardiological follow-up and an appropriate control of risk factors can improve the evolution of these patients.

## Figures and Tables

**Table 1:** Class of hormonal drugs used in advanced prostate cancer.

Class of Drugs	Examples
GnRH agonist ^a^	Leuprolide, Buserelin, Goserelin, Triptorelin
GnRH antagonist ^b^	Degarelix, Relugorix
Cytochrome P450-17 A1 inhibitor	Abiraterone
Nonsteroidal androgen receptor antagonists	Bicalutamide, Flutamide, Nilutamide, Enzalutamide, Apalutamide, Darolutamide
5α-reductase inhibitors	Finasteride, Dutasteride

^a^ GnRH = gonadotropin releasing hormone receptor agonist; ^b^ GnRH antagonist = gonadotropin releasing hormone receptor antagonist.

**Table 2:** Inclusion criteria for the current narrative review.

Population	Adult Men with Prostate Cancer
Intervention	Androgen deprivation therapy
Comparisons	Placebo, no therapy, radiotherapy, orchiectomy, watchful waiting/active surveillance, local therapy
Outcomes	Hypertension, myocardial infarction, stroke, arrhythmia, heart failure, venous thromboembolism, QT interval prolongation, sudden cardiac death, cardiovascular mortality

**Table 3:** Studies regarding the cardio-vascular effects of the androgen deprivation therapy.

First Author [Reference]	Year	Study Type	Pts Enrolled	Type of ADT/No pts	Treatment in the Control Group/No pts	Median Age	Follow-Up	Results
Studies Showing an Additional Cardio-Vascular Risk in Patients on ADT								
Keating [53]	2006	Observational SEER program 11 population-based cancer registries	73,193	GnRH agonists 25,570	No therapy/orchiectomy 47,623	74.2 ± 5.8	4.5 years	Increased risk of DM, CAD, MI, SCD, in the GnRH agonist group versus non therapy group Increased risk of DM in orchiectomy group versus non therapy group
D’Amico [54]	2007	Randomized study	1372	GnRH agonist + flutamide + RT	RT	70.88	3–8 months	Fatal MI occurs earlier in men older than 65 years who received 6 months of ADT than in those without ADT and in men younger than 65 years. No significant difference (*p* = 0.97) was observed in the time to fatal MIs in men age 65 years or older who received 6 to 8 months of ADT compared with 3 months of ADT
Tsai [55]	2007	Observational study	4881	3262 pts on radical prostatectomy + ADT in 1051 pts	RT 1630 pts	63	3.8 years	The use of ADT appears to be associated with an increased risk of death from cardiovascular causes in patients undergoing radical prostatectomy for localized prostate cancer
O’Farrell [52]	2015	Retrospective study	229,147	41,362 pts on GnRH agonist ± non steroidian antiandrogen	187,785 cancer free pts	75	6 years	CVD risk was highest during the first 6 months of ADT in men who experienced two or more cardiovascular events before therapy
Alibhai [56]	2009	Cohort study	116769	46,995 on ADT	No ADT 69,774	75 ± 6.3	6.47 years	ADT use for at least 6 months in older men is associated with an increased risk of diabetes and fragility fracture but not MI or sudden cardiac death.
Studies Showing No Additional Cardio-Vascular Risk in Patients on ADT								
Nanda [57]	2009	Retrospective study	5077	1521 pts on GnRH agonist+ non steroidian antiandrogen	3556 RT	69.5	2 years	ADT is significantly associated with an increased risk of all-cause mortality among men with a history of CAD-induced HF or MI but not among men with no comorbidity or a single CAD risk factor.
Bolla [58]	2009	Randomized study	970	487 pts on RT + 2.5 years GnRH agonists	483 pts on RT + 6 months GnRH agonists	69	6.4	There was no significant difference in the cumulative incidence of fatal cardiac events at 5 years: 4.0% in the short-term group and 3.0% in the long-term group
Punnen [59]	2011	Retrospective study of CaPSURE registry	7248	ADT (1086 pts) ADT + local therapy (485 pts)	Local therapy (5170 pts), watchful waiting/active surveillance (506 pts)	>65	47.6–57 months	A propensity-matching algorithm in a subset of 1391 patients was unable to find a significant difference in cardiovascular mortality between those who did or did not receive ADT.
Efstathiou [60]	1987–1992	randomized	945	RT + goserelin (477)	RT (468)	70	9 years	GnRH agonists do not seem to increase cardiovascular mortality in men with locally advanced prostate cancer
Studies That Compared Different Type of ADT Regarding Their Cardiotoxicity								
Liang [61]	2009–2017	Meta analysis	5 studies	ADT 63,258	Non ADT 209,403	69–75		Abiraterone and enzalutamide increased risk of AMI, CAD, in contrast, this association is not detected in SCD.
Shore [62]	2017–2018	Randomized phase III trial (HERO)	930 pts	622 pts relugolix	308 pts leuprolide	71	1 year	Relugolix achieved rapid, sustained suppression of testosterone levels that was superior to that with leuprolide, with a 54% lower risk of major adverse cardiovascular events
Moreira [63]	2011–2014	Meta-analysis	4 studies/5183 pts	Abiraterone-prednisone/prednisone 1333/936	Enzalutamid/placebo 1672/1244	46–95	5 years	Abiraterone was associated with cardiovascular adverse effects; enzalutamide was associated with fatigue
Margel [64]	2019	Phase II randomized study included patients with pre-existing cardio-vascular disease; primary endpoint endothelial dysfunction	80 pts	GnRH antagonist/41 pts	Gn agonist/39 pts	71–72	1 year	20% randomized to GnRH agonist experienced a major cardiovascular and cerebrovascular event compared to 3% of those on GnRH antagonist
Zhang [65]	2000–2020	Analysis of FDA Adverse Event Reporting System (FAERS}		6231 hormone monotherapy	1793 combined therapy	≥18 years	20 years	GnRH antagonists were associated with fewer cardiovascular adverse events than GnRH agonists as monotherapy and combination therapy, especially in men ≥ 60 years

SEER = Surveillance Epidemiology and End Results, ADT = androgen deprivation therapy RT = radiotherapy GnRHagonist = gonadotrophin releasing hormone receptor agonist CAD = coronary artery disease MI = myocardial infarction; HF = heart failure CVD = cardiovascular disease DM = diabetes mellitus; SCD = sudden cardiac death.
